# CMTM Family Genes Affect Prognosis and Modulate Immunocytes Infiltration in Grade II/III Glioma Patients by Influencing the Tumor Immune Landscape and Activating Associated Immunosuppressing Pathways

**DOI:** 10.3389/fcell.2022.740822

**Published:** 2022-02-17

**Authors:** Zeyu Wang, Jingwei Zhang, Hao Zhang, Ziyu Dai, Xisong Liang, Shuwang Li, Renjun Peng, Xun Zhang, Fangkun Liu, Zhixiong Liu, Kui Yang, Quan Cheng

**Affiliations:** ^1^ Department of Neurosurgery, Xiangya Hospital, Central South University, Changsha, China; ^2^ National Clinical Research Center for Geriatric Disorders, Changsha, China; ^3^ Clinical Diagnosis and Therapy Center for Glioma of Xiangya Hospital, Central South University, Changsha, China

**Keywords:** CMTM, lower-grade glioma, micro-environment, immune infiltration, prognosis

## Abstract

Lower-grade glioma (LGG) is one of the most common primary tumor types in adults. The chemokine-like factor (CKLF)-like Marvel transmembrane domain-containing (CMTM) family is widely expressed in the immune system and can modulate tumor progression. However, the role of the CMTM family in LGG remains unknown. A total of 508 LGG patients from The Cancer Genome Atlas (TCGA) database were used as a training cohort, and 155 LGG patients from the Chinese Glioma Genome Atlas (CGGA) array database, 142 LGG patients from the CGGA RNA-sequencing database, and 168 LGG patients from the GSE108474 database were used as the validation cohorts. Patients were subdivided into two groups using consensus clustering. The ENET algorithm was applied to build a scoring model based on the cluster model. Finally, ESTIMATE, CIBERSORT, and xCell algorithms were performed to define the tumor immune landscape. The expression levels of the CMTM family genes were associated with glioma grades and isocitrate dehydrogenase (IDH) status. Patients in cluster 2 and the high-risk score group exhibited a poor prognosis and were enriched with higher grade, wild-type IDH (IDH-WT), 1p19q non-codeletion, MGMT promoter unmethylation, and IDH-WT subtype. Patients in cluster 1 and low-risk score group were associated with high tumor purity and reduced immune cell infiltration. Enrichment pathways analysis indicated that several essential pathways involved in tumor progression were associated with the expression of CMTM family genes. Importantly, PD-1, PD-L1, and PD-L2 expression levels were increased in cluster 2 and high-risk groups. Therefore, the CMTM family contributes to LGG progression through modulating tumor immune landscape.

## Introduction

Gliomas originate from the neuroglial stem or progenitor cells and is the most common primary malignant brain tumor (Weller et al., 2015). Gliomas are classified into two categories according to the degree of malignancy, including glioblastoma (GBM) and lower-grade glioma (LGG). In addition, 2016 WHO classification of the central nervous system (CNS) tumors classified gliomas into astrocytic tumors, oligodendrogliomas, and not otherwise specified (NOS) not only based on histology but also molecular features, including isocitrate dehydrogenase (IDH) mutational and 1p/19q codeletion status (Wen and Huse, 2016; Wen and Huse, 2017). LGG has a relatively long growth period in adults. Therefore, surgical management, radiotherapy, and chemotherapy are the main treatments for LGG (Kleihues and Ohgaki, 1999; Taylor et al., 2019). Although people with LGG have a better prognosis than other aggressive tumor types in the CNS, the median overall survival (OS) of LGG is still far from satisfactory (Sidaway, 2020; Aiman and Rayi, 2021).

In recent years, numerous studies have focused on the immune infiltration of glioma. A close relationship has been shown between tumor-infiltrating immune cells and improved clinical outcomes in LGG (Bacolod et al., 2016; Wu et al., 2019a; Zhang et al., 2021a). The tumor microenvironment (TME) is the environment around a tumor that includes the surrounding blood vessels, infiltrated immune cells, tumor cells, cytokines, and the extracellular matrix (ECM) (Giraldo et al., 2019). TME is a dynamic and complex ecosystem that mediates tumor immunity, tumorigenesis, tumor growth, and migration. Microglia, macrophages, regulatory T cells (Tregs), and natural killer cells (NK) are the primary immune cells in the TME (Binnewies et al., 2018). These immune cells interact with tumor cells and play an essential role in modulating multiple immune processes in the TME. Importantly, they mediate the progress of tumor growth and metastasis. For example, CD8^+^ T cells and CD4^+^ T-helper 1 (Th1)-oriented T cells can directly kill tumor cells in an antigen-specific manner by secreting cytotoxic cytokines (Lim et al., 2020). Conversely, other immune cells, such as the M2 type of macrophages, express C-C motif chemokine receptor 2 (CCR2, receptor of CCL2), and which is an irreplaceable factor that promotes tumor metastasis. Inhibition of the CCL2-CCR2 pathway can effectively reduce tumor metastasis and eventually prolong the survival of mice (Qian et al., 2011). Therefore, TME-specific immunotherapy may provide promising targets in the treatment of LGG in the future.

The CKLF-like Marvel transmembrane domain-containing (CMTM) gene, which contains nine members (CMTM1-8 and CKLF), is a novel gene family reported in 2001 (Han et al., 2001). CMTM members are widely expressed in the immune system and are involved in several pathological processes (Delic et al., 2015). A recent study found that abnormal expression of CMTMs was implicated in the process of tumor growth and metastasis (Wu et al., 2019b; Wu et al., 2020). However, the specific role and mechanism remain elusive. Herein, we used clinical, mRNA sequencing, and microarray data of LGG patients from The Cancer Genome Atlas (TCGA), and Chinese Glioma Genome Atlas (CGGA) databases, and Gene Expression Omnibus (GEO) database (Bao et al., 2014; Fang et al., 2017; Zhao et al., 2017) to evaluate the predictive value of the CMTM family in LGG and elucidate the relationship between the CMTM family genes and immune infiltration in LGG TME.

## Materials and Methods

### Datasets

The clinical and expression data of LGG patients were collected from TCGA database (http://cancergenome.nih.gov/) and CGGA database (http://www.cgga.org.cn). Data from TCGA (*n* = 508) were used as the training cohort and CGGA array data (CGGA301, mRNA microarray database, *n* = 155), CGGA RNA-sequencing (RNA-seq) data (CGGA325, mRNA sequencing database, *n* = 142), and GSE108474 data (*n* = 168) were used as validation cohorts. The LGG subtype was predicted by the Gliovis data portal (Bowman et al., 2017).

### Consensus Clustering Analysis and Machine Learning

Consensus clustering analysis based on CMTMs was performed with the R package “ConsensusClusterPlus” (Wilkerson and Hayes, 2010). For the validation cohort, machine learning, support vector machine, was used to reproduce the clustering model with the R package “e1071”. The heatmap and Sankey diagram were generated to illustrate the potential relationship between LGG clinical features and the clustering model.

### Construction of the Risk Model

Differentially expressed genes (DEGs) between two clusters were identified with the R package ‘limma’. Univariate Cox regression analysis and the ENET algorithm were further employed to filter out prognosis-associated genes. Finally, the risk was calculated based on principal component analysis (PCA) and hazard ratio (HR):

Risk=Gene(HR>1)*(PC1+PC2)−Gene(HR<1)*(PC1+PC2) (2)

### Biofunction Prediction

Gene ontology (GO) and Kyoto Encyclopedia of genes and genomes (KEGG) based on gene set variation analysis (GSVA) and gene set enrichment analysis (GSEA) were used to explore differential biofunction prediction activation between cluster 1 and cluster 2 samples, as well as samples in the high-risk and low-risk groups. Pathways with *p* value <0.05 are considered as statistically significant.

### Tumor Immune Landscape

The landscape of immune cell infiltration was predicted with the R package ‘ESTIMATE’ (Yoshihara et al., 2013), CIBSERSORT algorithm (Newman et al., 2019), and xCell algorithm (Aran et al., 2017) as previously described.

### Univariate and Multivariate Cox Regression Analysis

Univariate and multiple variate Cox regression analysis were performed with R package “survival”, and results were presented with R package “forestplot”.

### Real-Time Quantitative PCR

Tumor tissue and normal tissue which was adherent to the tumor were collected during the surgery. One milliliter Trizol was added to 100 mg tissue, and tissue was grinded by a grinder (Servicebio, Wuhan, China). Then, RNA extraction was followed by the standard protocol. cDNA library was constructed following protocol of HiScript II Q RT SuperMix for qPCR (+g DNA wiper) (Vazyme, Nanjing, China). Steps of real-time quantitative PCR were set as previously reported (Wang et al., 2021), and primers are listed as below:

 β-actin
    Forward: ACC​CTG​AAG​TAC​CCC​ATC​GAG
      Reverse: AGC​ACA​GCC​TGG​ATA​GCA​AC
 CMTM3
    Forward: AAG​TAC​TCG​GAT​GGG​GCT​TC
      Reverse: TCT​TCT​GTC​TTG​TGG​GCT​GT
 CMTM6
    Forward: TTT​CCA​CAC​ATG​ACA​GGA​CTT​C
      Reverse: GGC​TTC​AGC​CCT​AGT​GGT​AT
 CMTM7
    Forward: CCA​AGA​GTT​ACA​ACC​AGA​GCG
      Reverse: CAT​CTG​TGG​ACT​GGG​TTA​CAC
 CMTM8
    Forward: AAC​AAT​GAC​CTA​CAC​CAG​GAT​TC
      Reverse: AAG​GCA​CTG​CCG​TTA​AAG​C
 CKLF
    Forward: CAC​AAG​CCC​CTG​AAC​CAT​AT
      Reverse: GCT​TCC​GGT​AAA​TAA​GGG​CC

### Statistical Analysis

The Wilcox test was used to compare the difference between two groups, and analysis of variance (ANOVA) was used to compare multiple groups. In addition, the Kaplan-Meier log-rank test was used for survival analyses. All analyses were performed with R (version 3.6.1), and GraphPad Prism (version 8.0.1).

## Results

### Relationship Between CMTM Family Genes and Tumor Grade and IDH Status

**FIGURE 1:**
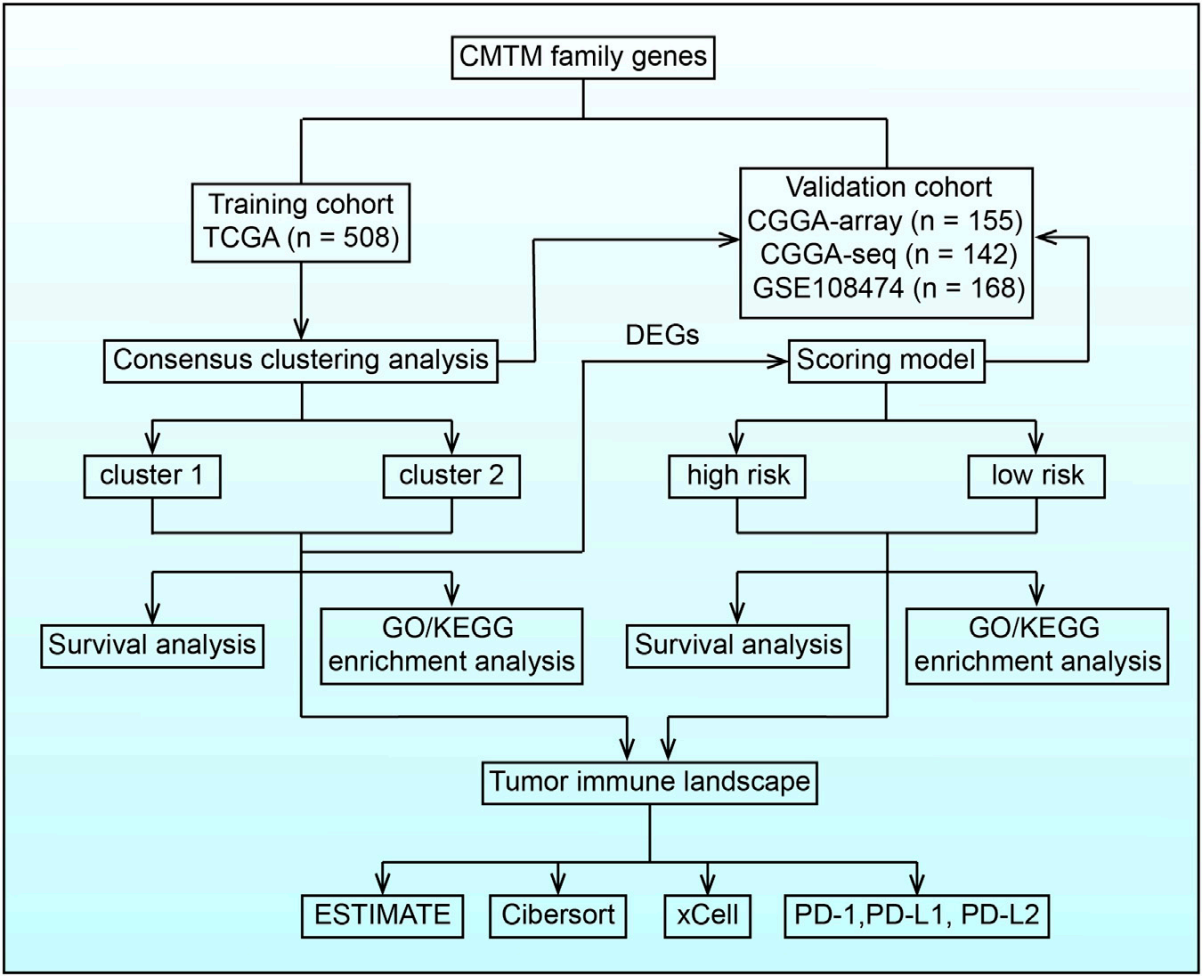
Flow chart illustrating the proposed role of CMTM family genes in LGG.

**FIGURE 2:**
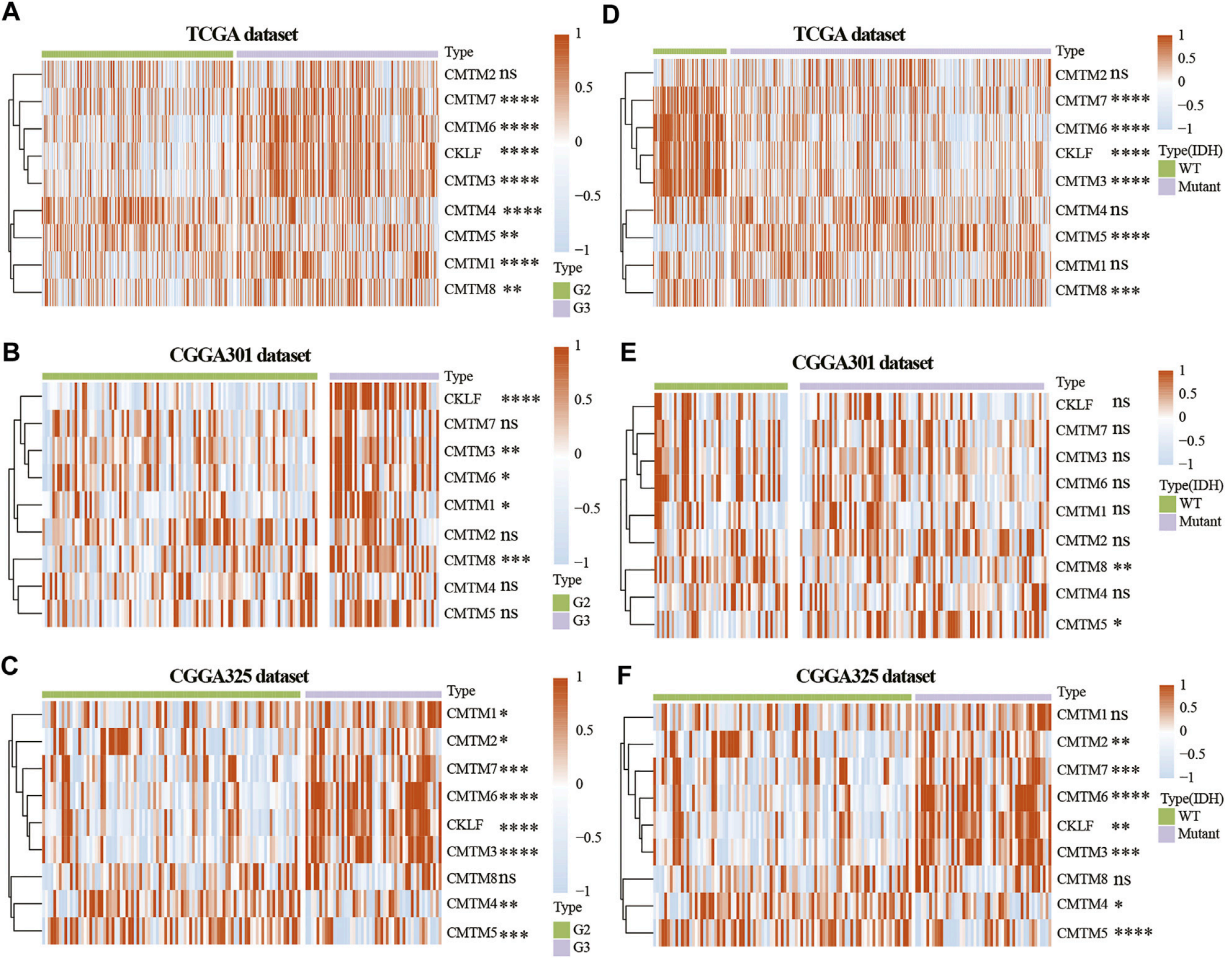
Expression of CMTM family genes in LGG in training and validation cohorts. Expression of CMTM family genes in different grades in the TCGA **(A)**, CGGA301 **(B)**, and CGGA325 **(C)** datasets. Expression of CMTM family genes in different IDH subgroups in the TCGA **(D)**, CGGA301 **(E)**, and CGGA325 **(F)** datasets. The Wilcox test was used to compare the difference between two groups (ns: no significant difference, *p < 0.05, **p < 0.01, ***p < 0.001, and ****p < 0.0001).

To study the role of CMTM family genes in LGG, LGG data were selected from TCGA, CGGA array (CGGA301), and CGGA RNA-seq (CGGA325) databases (Figure 1). The description of samples from the TCGA, CGGA-seq, CGGA-array, and GSE108474 databases were shown in Supplementary Table S1. The relationship between the expression levels of each CMTM family gene and grade from the TCGA database (training cohort) was explored. Results showed that the expression level of CMTM1, CMTM3, CMTM6, CMTM7, CMTM8, and CKLF was elevated in high-grade gliomas (grade III; *p* < 0.01; Figure 2A). Conversely, the expression level of CMTM4 and CMTM5 was higher in LGG (grade II; *p* < 0.01; Figure 2A). Similarly, the expression levels of CMTM1, CMTM3, CMTM6, CMTM8, and CKLF were increased in grade III in the validation cohort (CGGA301; *p* < 0.05; Figure 2B). IDH mutational status is another critical marker that is related to patient prognosis. The result indicated that the levels of CMTM3, CMTM6, CMTM7, CMTM8, and CKLF were much higher in IDH-WT than in IDH-mutated gliomas in the TCGA database (*p* < 0.001; Figure 2D). Data from the CGGA301 database showed that only CMTM8 was upregulated in IDH-WT compared with the IDH mutational group (*p* < 0.01; Figure 2E), which could be due to fewer samples in CGGA301 than TCGA database. The expression level of CMTM5 was lower in IDH-WT gliomas in both TCGA and CGGA301 databases. Moreover, data from CGGA325 showed that whereas the mRNA levels of CMTM2, CMTM3, CMTM6, CMTM7, and CKLF were significantly elevated in both grade III and IDH-mutant groups, CMTM4 and CMTM5 expression levels were decreased in both grade III and IDH WT groups (all *p* < 0.05; Figures 2C,F). Overall, these results indicated that high levels of CMTM3, CMTM6, CMTM7, and CKLF might predicat a worse prognosis, while the high levels of CMTM4 and CMTM5 may be associated with favorable outcomes in LGG.

The expression of CMTM3, CMTM6, CMTM7, CMTM8, and CKLF were further verified on our own samples (Supplementary Figure S1). As illustrated, the expression of CMTM3, CMTM6, CMTM7, CMTM8, and CKLF in Grade II and III gliomas showed similar tendency as that in TCGA and CGGA databases.

### Relationship Between Clusters of CMTM Family Genes and Glioma Prognosis

**FIGURE 3:**
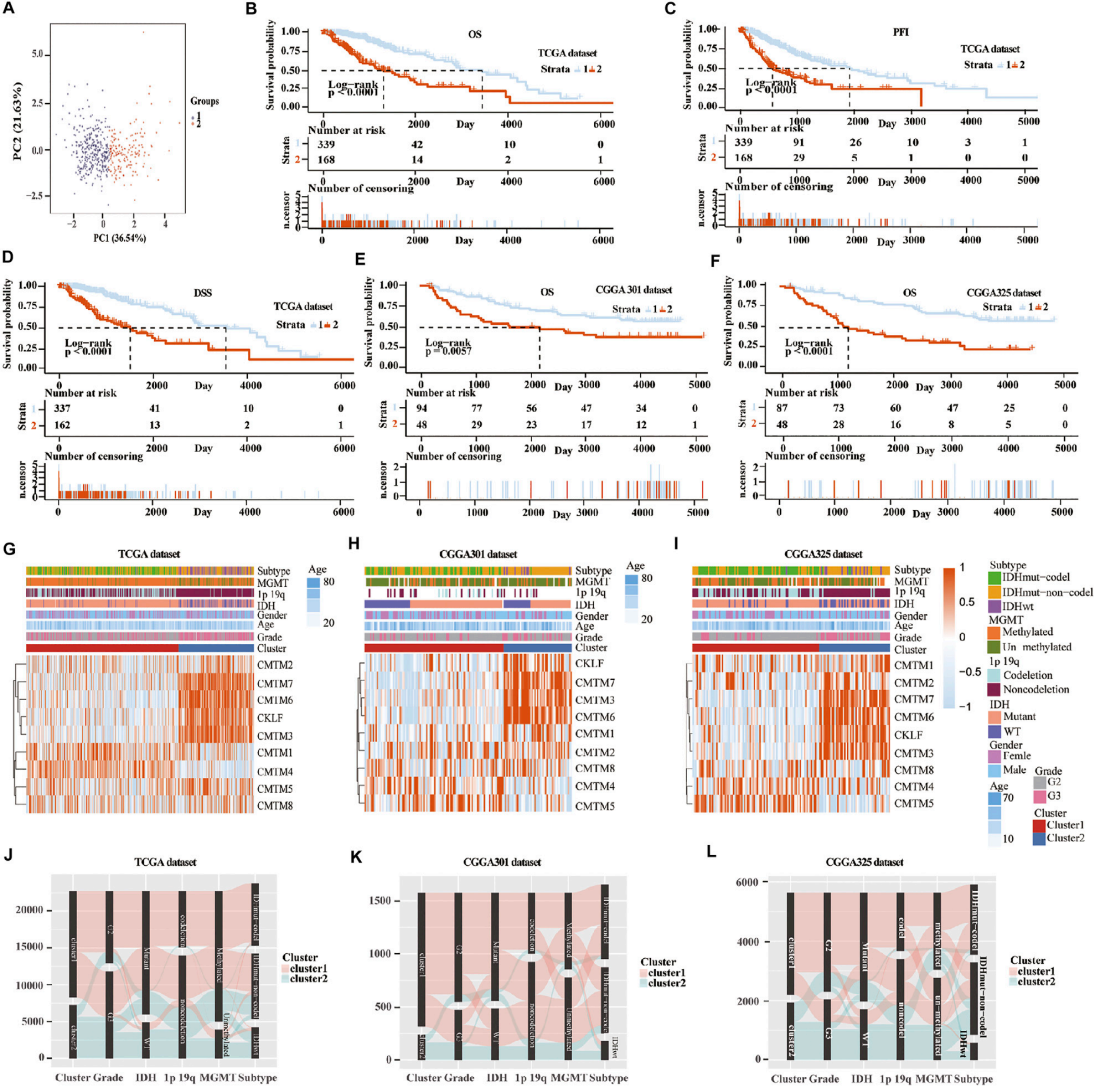
Consensus clustering of samples into cluster 1 and cluster 2 in training and validation cohorts. **(A)** Principal component analysis (PCA) was applied to explore any clustering behavior of the samples in the TCGA database. Kaplan-Meier analysis of patients in cluster 1 and cluster 2 from the TCGA database for OS **(B)**, PFI **(C)**, and DSS **(D)**. (E) Kaplan-Meier analysis of patients in cluster 1 and cluster 2 from CGGA301 database for OS. **(F)** Kaplan-Meier analysis of patients in cluster 1 and cluster 2 from CGGA325 database for OS. Heatmap of different gliomas features and expression of CMTM in cluster 1 and cluster 2 from TCGA **(G)**, CGGA301 **(H)**, and CGGA325 **(I)** databases. Sankey diagram of different expression patterns of glioma features in cluster 1 and cluster 2 from TCGA **(J)**, CGGA301 **(K)**, and CGGA325 **(L)** databases, analyzed by the log-rank test.

Unsupervised consensus clustering was used to classify patients into cluster 1 and cluster 2 in the training and validation cohorts. First, machine learning was used to reproduce the clustering model in the TCGA cohort (Figure 3A). Then, Kaplan-Meier analysis was used to reveal the outcome in the two clusters. Result in the TCGA database showed that patients in cluster 1 had a better prognosis compared with those in cluster 2 for OS, progression-free interval (PFI), and disease-specific survival (DSS) (*p* < 0.0001; Figures 3B–D). In the three validation cohorts, patients in cluster 1 also showed a better outcome for OS (*p* < 0.01; Figures 3E,F; Supplementary Figure S5A). The correlation between CMTM family expression, the cluster model, and corresponding clinical features were also mapped in TCGA (Figure 3G), CGGA-array database (Figure 3H), and CGGA RNA-sequence database (Figure 3I). Furthermore, the Sankey diagram from the TCGA database showed that numerous factors, including higher grade (grade III), IDH-WT, 1p/19q non-codeletion, and O6-methylguanine-DNA methyltransferase (MGMT) unmethylation, were associated with poor prognoses in cluster 2 (Figure 3J), which were verified in CGGA-array (Figure 3K) and CGGA RNA-seq (Figure 3L) databases. These results indicated that the expression of CMTM family genes was associated with patient prognosis in LGG.

### Relationship Between CMTM Family Genes and Immune Cell Infiltration in LGG Microenvironment

**FIGURE 4:**
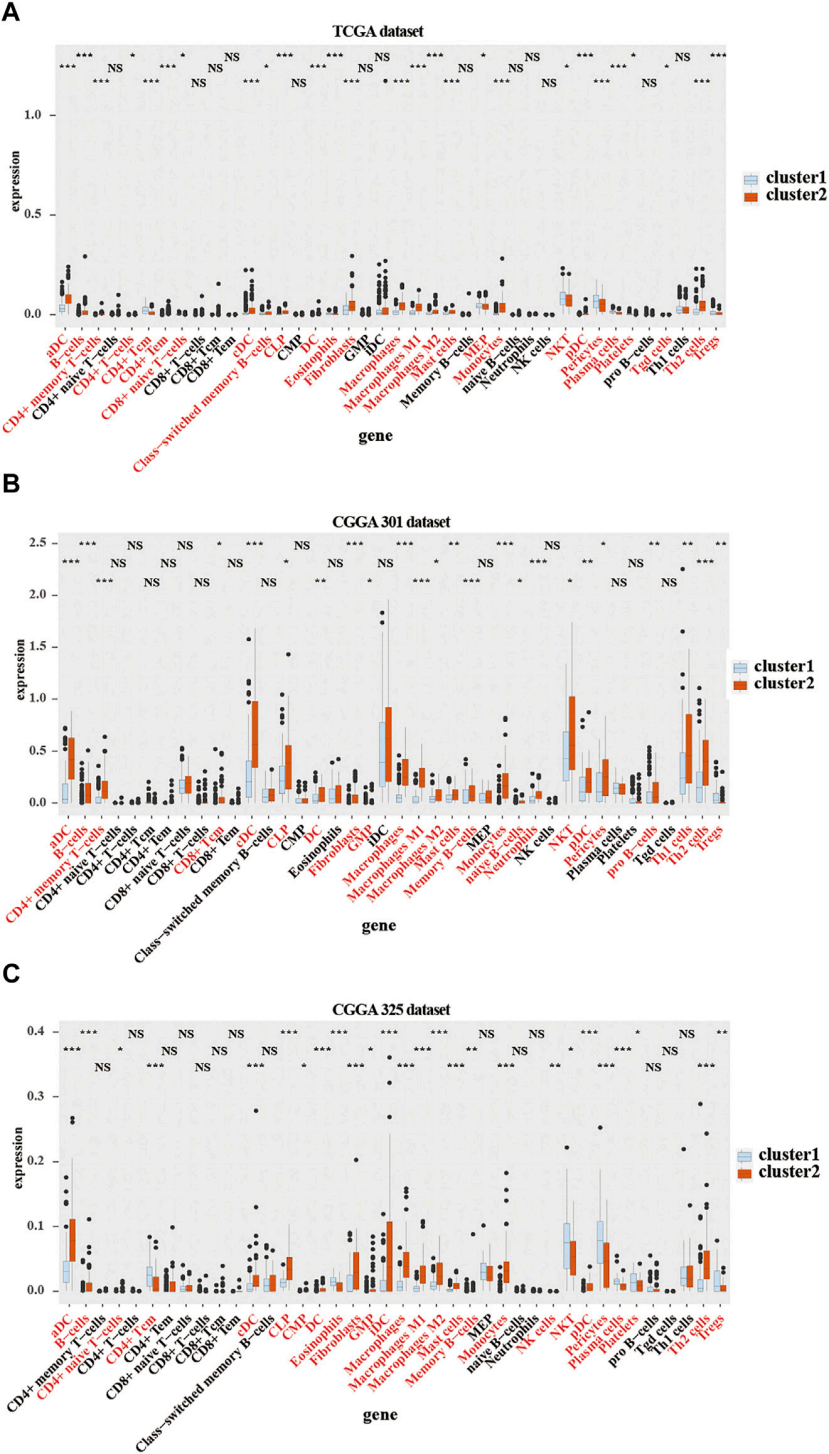
Immune cells infiltration in cluster 1 and cluster 2 from TCGA and CGGA301 databases. xCELL algorithm of infiltrated immune cells in cluster 1 and cluster 2 from TCGA **(A)**, CGGA301 **(B)**, and CGGA301 **(C)** databases. The Wilcox test was used to compare the difference between the two groups (ns: no significant differences, *p < 0.05, **p < 0.01, and ***p < 0.001).

To further verify the relationship between the expression of CMTM family genes and immune cell infiltrates in the LGG microenvironment, the purity, ESTIMATE score, stromal score, and immune score were calculated in the two clusters in training and validation cohorts. ESTIMATE, stromal, and immune scores were higher in cluster 2 (*p* < 0.001), whereas tumor purity was lower (*p* < 0.001) in cluster 2 in training and validation cohorts (Supplementary Figure S2). Moreover, results from the xCell algorithm showed different immune cell infiltrates in cluster 1 and cluster 2 in training and validation cohorts (Figures 4A–C). The expression levels of dendritic cells (DCs), B cells, common lymphoid progenitor cells (CLPs), fibroblasts, macrophages (M1 and M2), mast cells, Th2 cells, and monocytes were significantly higher in cluster 2 in the three cohorts (*p* < 0.05). Contrarily, the expression levels of natural killer T (NKT) cells, and regulatory T cells (Tregs) were higher (*p* < 0.05) in cluster 1. Results from the CIBERSORT algorithm indicated that the levels of M1 and M2 macrophages were significantly higher in cluster 2 (*p* < 0.01; Supplementary Figure S3). However, the expression of CD4 naive T cells was higher in cluster 1 (*p* < 0.001). Collectively, these results revealed that many immune infiltrating cells were closely associated with the poor prognosis of patients in cluster 2.

### Functional Enrichment Analysis in Clusters 1 and 2

**FIGURE 5:**
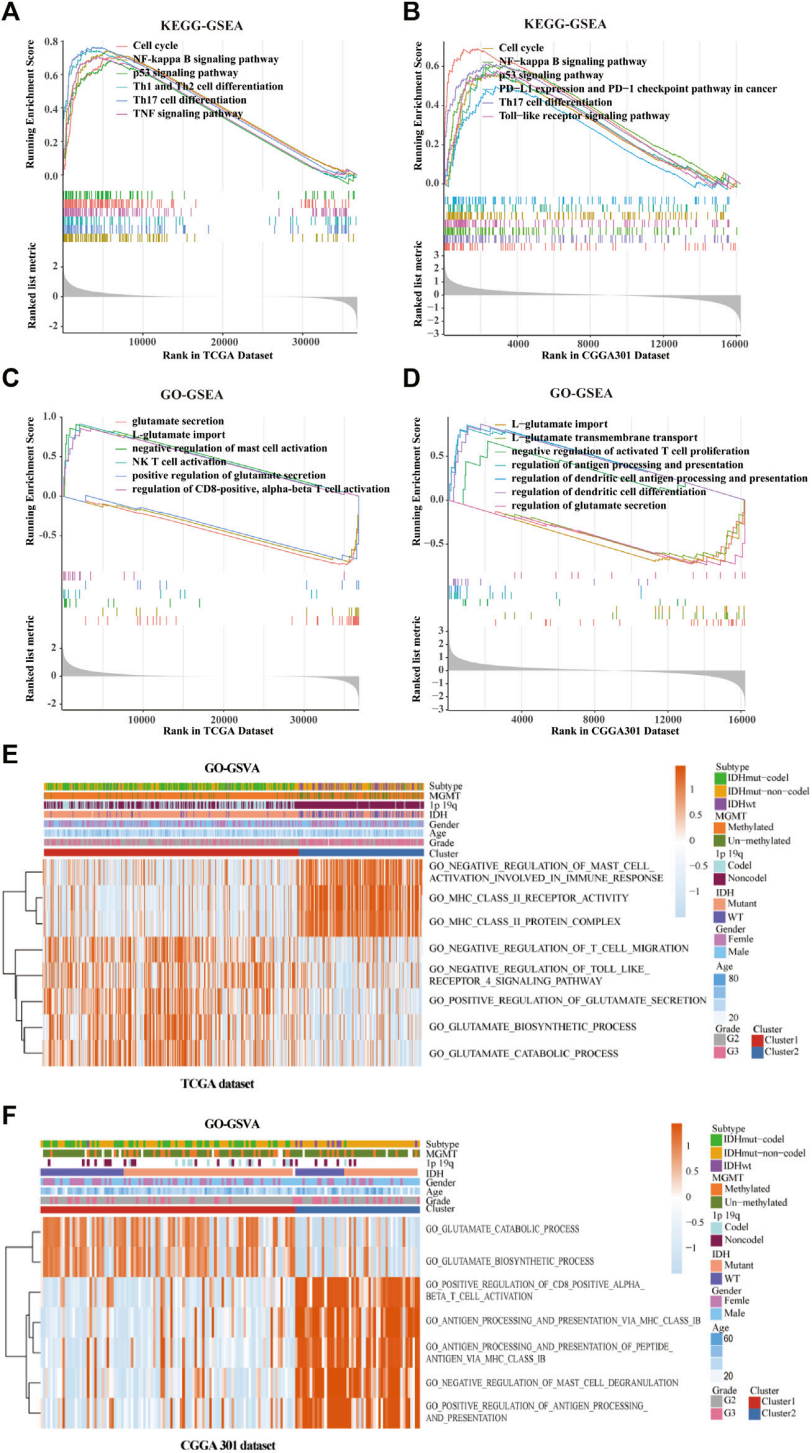
Gene ontology (GO) and Kyoto Encyclopedia of Genes and Genomes (KEGG) enrichment analysis of the cluster model based on gene set enrichment analysis (GSEA) and gene set variation analysis (GSVA). Enriched pathways between cluster 1 and cluster 2 based on KEGG-based GSEA analysis in TCGA **(A)** and CGGA301 **(B)** databases. Enriched pathways between cluster 1 and cluster 2 based on GO-based GSEA analysis in TCGA **(C)** and CGGA301 **(D)** databases. Enriched pathways between cluster 1 and cluster 2 based on GO-based GSVA analysis in TCGA **(E)** and CGGA301 **(F)** databases.

GO/KEGG enrichment analysis based on GSEA and GSVA analyses were used to identify differential pathways activation between cluster 1 and cluster 2 samples. KEGG-based GSEA pathway analysis in TCGA and CGGA-array databases indicated that pathways associated with p53 signaling, programmed death-ligand 1 (PD-L1) expression, programmed cell death protein 1 (PD-1) checkpoint, and Th17 cell differentiation were highly enriched (Figures 5A,B) in cluster 2 samples. GO-based GSEA pathway analysis in TCGA and CGGA-array databases indicated that pathways associated with L-glutamate importation and glutamate secretion were highly enriched (Figures 5C,D) in cluster 1 samples while immune related pathways like negative regulation of mast cell activation were enriched in cluster 2 samples. Data from the CGGA RNA-seq cohort also supported those difference (Supplementary Figures S4A,B). Moreover, KEGG-based GSVA analysis, visualized by heatmap, showed different pathways in cluster 1 and cluster 2. In the TCGA database, pathways associated with negative regulation of mast cell activation, major histocompatibility complex (MHC) class Ⅱ receptor activation, and protein complex were enriched in cluster 2. In addition, pathways associated with negative regulation of T cell migration, adverse effects of Toll-like receptor 4 (TLR4) signaling, positive regulation of glutamate secretion, glutamate biosynthetic process, and glutamate catabolic process were enriched in cluster 1 (*p* < 0.05; Figure 5E). In the CGGA-array dataset, the glutamate bio-synthetic and catabolic process pathways were enriched in cluster 1 (*p* < 0.05). However, pathways associated with CD8^+^ αβ T cell activation, antigen progress and presentation via MHC class Ⅰ B cell, negative regulation of mast cells degranulation, and positive regulation of antigen progress and expression were enriched in cluster 2 (*p* < 0.05; Figure 5F). Similar results were also found in the CGGA RNA-seq dataset (Supplementary Figure S4C).

### Establishment of the Scoring Model Based on DEGs in the Training Cohort

**FIGURE 6:**
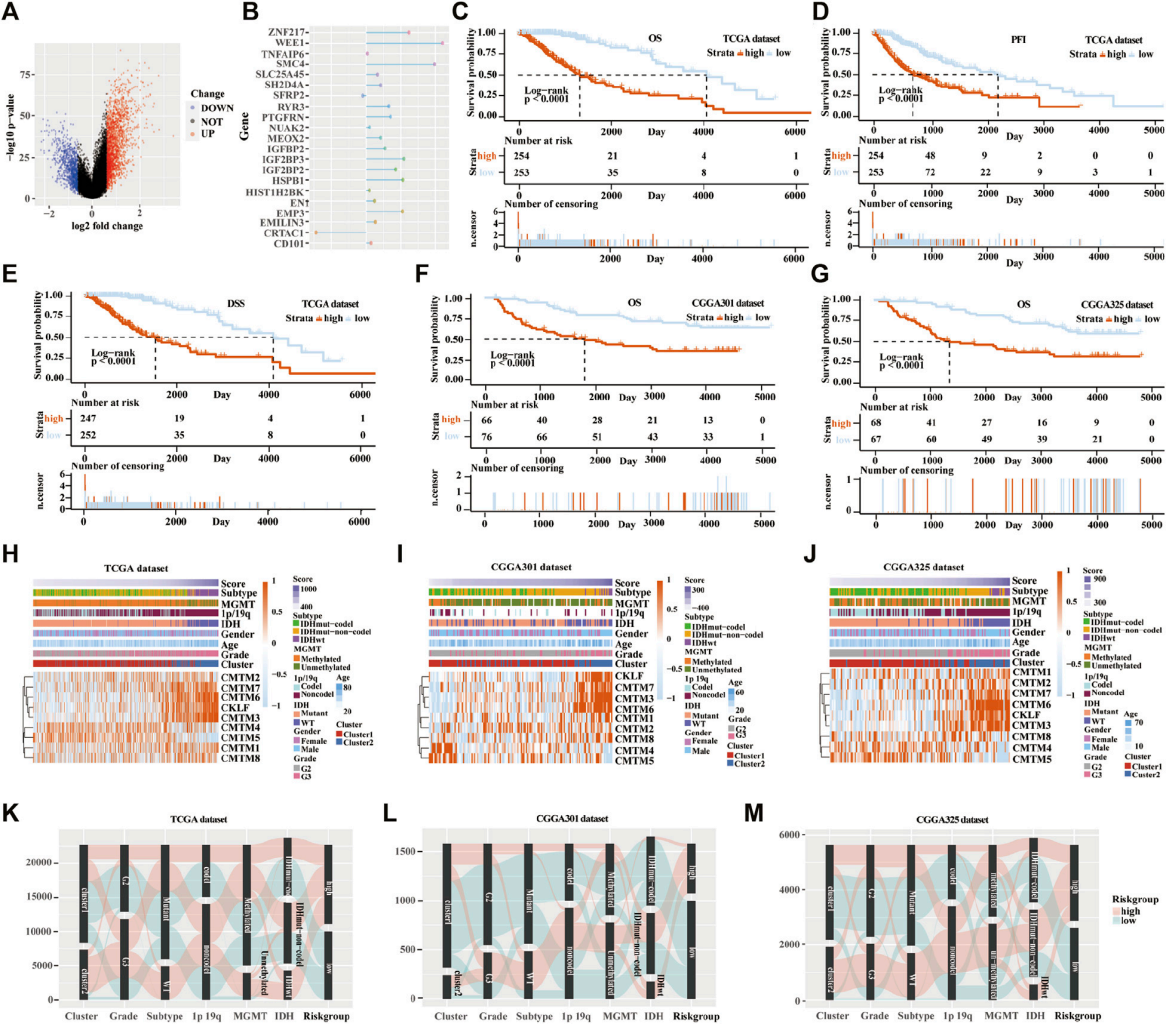
Establishment and verification of the scoring model in the training and validation cohorts. **(A)** Volcano plot of differently expressed genes in the two clusters from TCGA database. **(B)** Elastic network map of 21 genes that were associated with prognosis in the two clusters from the TCGA database. Kaplan-Meier analysis of patients in low-risk and high-risk groups from the TCGA database for OS **(C)**, PFI **(D)**, and DSS **(E)**. **(F)** Kaplan-Meier analysis of patients in low-risk and high-risk groups from the CGGA301 database for OS. **(G)** Kaplan-Meier analysis of patients in low-risk and high-risk groups from the CGGA325 database for OS. Heatmap of different glioma features and expression of CMTM in the low-risk and high-risk groups from the TCGA **(H)**, CGGA301 **(I)**, and CGGA325 **(J)** databases. Sankey diagram of different expression patterns of glioma features in cluster 1 and cluster 2 from TCGA **(K)**, CGGA301 **(L)**, and CGGA325 **(M)** databases, analyzed by the log-rank test.

Next, the scoring models (low-risk and high-risk groups) were constructed based on the DEGs from clusters 1 and 2 based on TCGA database (Figure 6A). Then, a total of 21 genes were identified as being independently associated with prognosis in the TCGA database by exploring the univariate Cox regression analysis and the ENET algorithm subsequently (Figure 6B). The risk score was calculated based on those genes. Univariate and multivariate Cox regression analysis in TCGA, CGGA RNA-seq, CGGA-array, and GSE108474 databases were shown in Supplementary Table S2. Kaplan-Meier analysis was used to evaluate survival differences between the two risk groups. It was found that patients in the low-risk group had a better prognosis compared with those in the high-risk group for OS, PFI, and DSS in the TCGA database (*p* < 0.0001; Figures 6C–E). In addition, patients in the low-risk group had a better outcome for OS in the three validation cohorts (*p* < 0.0001; Figures 6F,G; Supplementary Figure S5B).

From the heatmap of the scoring models, high levels of CMTM3, CMTM6, CMTM7, and CKLF were associated with increased risk scores in TCGA and the two CGGA databases (Figures 6H–J; Supplementary Figure S5C). However, high levels of CMTM4 and CMTM5 were associated with a low-risk score. Glioma subtype, 1p/19q co-deletion, grade II gliomas, IDH mutational status, and MGMT methylation were associated with lower-risk scores in TCGA and the two CGGA databases (*p* < 0.001; Supplementary Figure S6). Furthermore, most samples in cluster 2 belonged to the high-risk group. The Sankey diagram showed that samples from cluster 1, lower grade (grade II), IDH mutational status, 1p/19q co-deletion, and MGMT methylation were associated with favorable prognoses in the low-risk group (Figures 6K–M).

### Functional Enrichment Analysis in the Low- and High-Risk Group in the Training and Validation Cohorts

**FIGURE 7:**
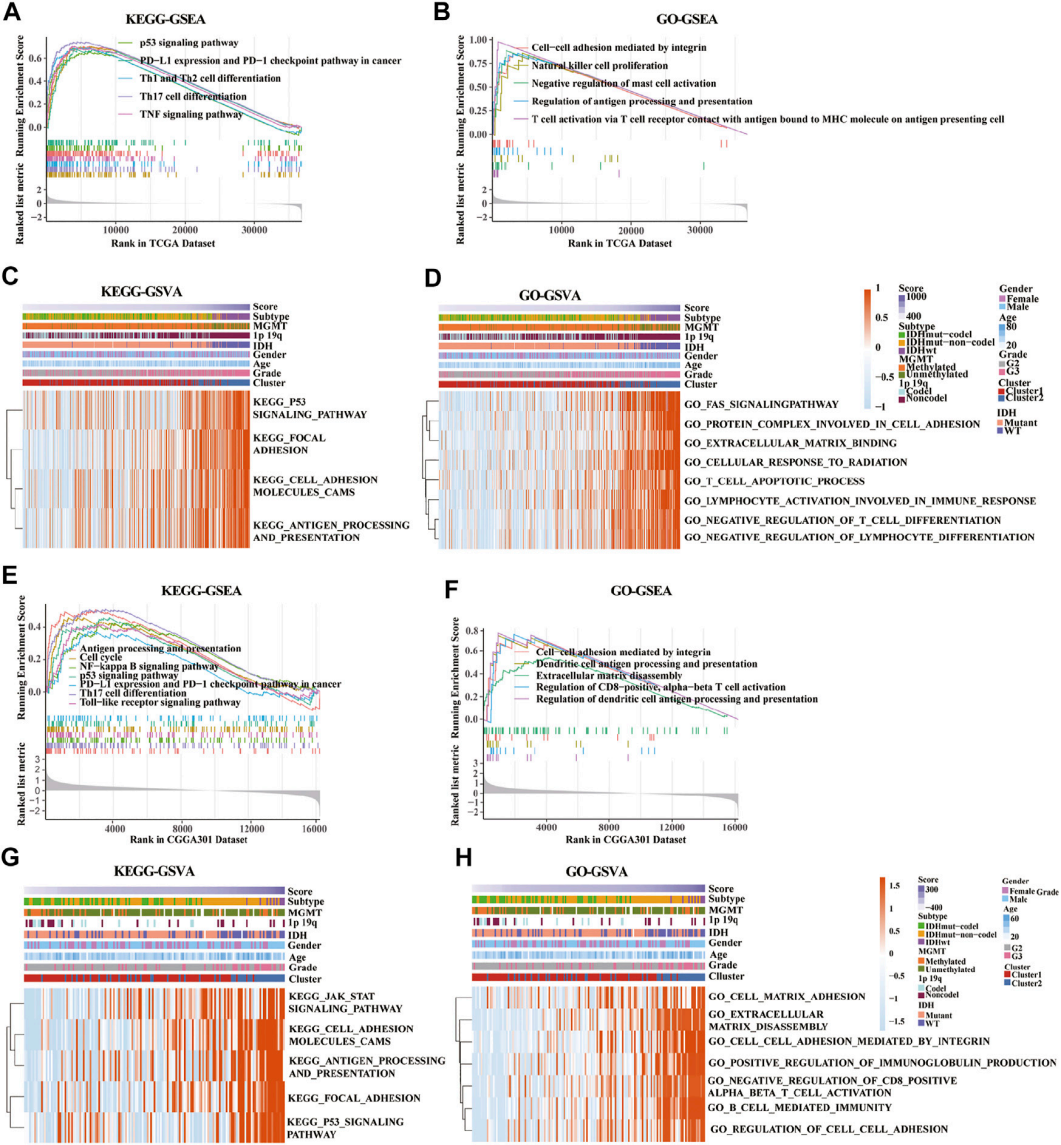
Gene set enrichment analysis (GSEA) and gene set variation analysis (GSVA) of key pathways between low-risk and high-risk groups based on gene ontology (GO) and Kyoto Encyclopedia of Genes and Genomes (KEGG) databases. **(A,E)** Enriched pathways between low-risk and high-risk groups based on KEGG-based GSEA analysis in TCGA **(A)** and CGGA301 **(E)** databases. **(B,F)** Enriched pathways between low-risk and high-risk groups based on GO-based GSEA analysis in TCGA **(B)** and CGGA301 **(F)** databases. **(C,G)** Enriched pathways between low-risk and high-risk groups based on KEGG-based GSVA analysis in TCGA **(C)** and CGGA301 **(G)** databases. **(D,H)** Enriched pathways between low-risk and high-risk groups based on GO-based GSVA analysis in TCGA **(D)** and CGGA301 **(H)** databases.

KEGG-based GSEA pathway analysis indicated p53 signaling, PD-L1 expression, PD-1 checkpoint, Th cell differentiation (Th1, Th2, and Th17), and TNF signaling were enriched in high-risk score group from the TCGA database (Figure 7A). GO-based GSEA pathway analysis indicated that cell-cell adhesion mediated by integrin, nature like cell proliferation, negative regulation of mast cell activation, restriction of antigen processing and presentation, and T cell activation pathways were also enriched in high-risk score group from the TCGA database (Figure 7B). Furthermore, KEGG-based GSVA analysis showed that the signaling pathways of p53, focal adhesion, cell adhesion molecules cams, antigen progress, and presentation were enriched in high-risk score group from the TCGA database (Figure 7C). In addition, GO-based GSVA analysis indicated that the signaling pathways of FAS, protein complex involved in cell adhesion, extracellular matrix binding, cellular response to radiation, cell apoptotic progress, lymphocyte activation involved in immune response, negative regulation of T cell, and lymphocyte differentiation were associated with the high-risk score in the TCGA database (Figure 7D).

Moreover, pathways associated with antigen process and presentation, NF-kappa B, p53 signaling, PD-L1 expression, PD-1 checkpoint, Th17 cell differentiation, and TLR signaling were enriched in high-risk score group from the CGGA-array database (Figure 7E). GO-based GSEA pathway analysis in the CGGA-array database indicated that cell-cell adhesion mediated by integrin, DC antigen processing and presentation, extracellular matrix disassembly, regulation of CD8^+^, and alpha-beta T cell activation signaling were enriched high-risk score group (Figure 7F). KEGG-based GSVA analysis showed that JAK/STAT signaling, cell adhesion molecules cams, antigen processing and presentation, focal adhesion, and p53 signaling pathways were enriched, with a high-risk score in high-risk score group from the CGGA-array database (Figure 7G). Finally, GO-based GSVA analysis indicated that signaling pathways associated with cell-matrix adhesion, extracellular matrix disassembly, cell-cell adhesion mediated by integrin, regulation of immunoglobulin production, cell-cell adhesion, CD8^+^, alpha-beta T cell activation, and cell-mediated immunity were enriched in high-risk score group from the CGGA-array database (Figure 7H). These results were also verified in the CGGA RNA-seq database (Supplementary Figure S7).

### Expression of PD-1, PD-L1, and PD-L2 in Different Clusters and Score Groups

**FIGURE 8:**
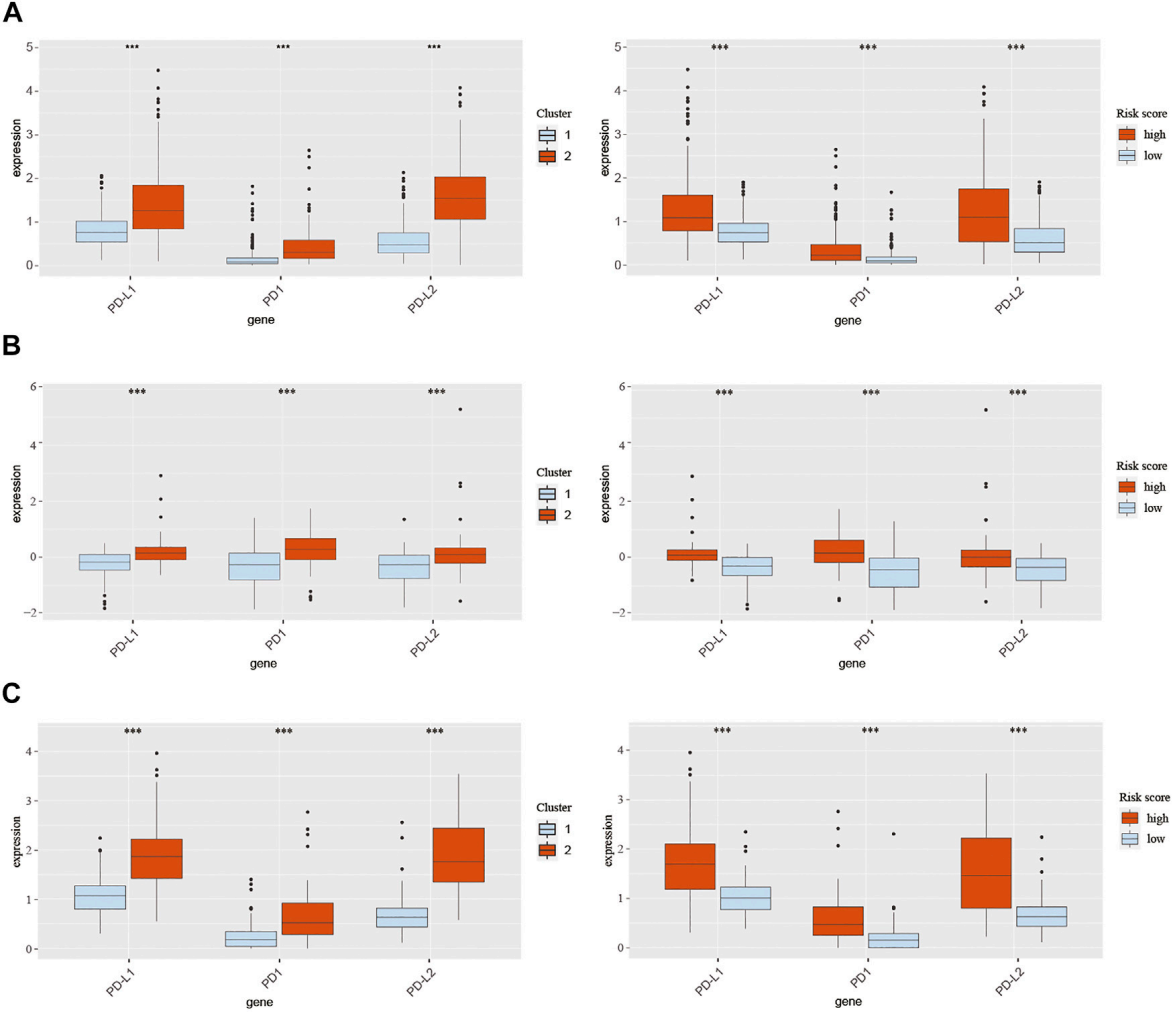
Expression of PD-1, PD-L1, and PD-L2 in different clusters and risk groups in training and validation cohorts. **(A)** Expression of PD-1, PD-L1, and PD-L2 in cluster 1, cluster 2, the low-risk group, and the high-risk group from TCGA database. **(B)** Expression of PD-1, PD-L1, and PD-L2 in cluster 1, cluster 2, the low-risk group, and the high-risk group from the CGGA301 database. **(C)** Expression of PD-1, PD-L1, and PD-L2 in cluster 1, cluster 2, the low-risk group, and the high-risk group from the CGGA325 database. The Wilcox test was used to compare the difference between the two groups (***p < 0.001).

Pathways enrichment analysis indicated that immunocytes’ function may be different between high and low score samples. Next, we explore the association between those two models and immune check point expression. The expression of CMTM family genes was previously found to be closely associated with immune checkpoints PD-1 and PD-L1. Therefore, PD-1, PD-L1, and PD-L2 expression levels were evaluated in different clusters and risk groups (Figure 8). Results indicated that the expression levels of PD-1, PD-L1, and PD-L2 were upregulated in cluster 2 and the high-risk group in all three cohorts (*p* < 0.001; Figures 8A–C). Together, those results implied an immunosuppressive microenvironment in high-risk samples.

## Discussion

LGG is a primary tumor that progresses slowly in the brain but eventually develops into high-grade secondary aggressive tumors, such as GBM (Bready and Placantonakis, 2019). The prognosis of LGG is affected by several factors, including age, grade, and molecular genetic mutations (IDH mutations, 1p/19q co-deletion, MGMT promoter methylation). IDH, a small molecular protein, is a key rate-limiting enzyme in the progress of energy metabolism. Numerous studies found that patients with IDH-mutated gliomas exhibited a better prognosis than those with IDH-WT (Hartmann et al., 2010; Turkalp et al., 2014). 1p/19q co-deletion is another valuable genetic marker for patients with gliomas. Zhao et al. evaluated 28 glioma studies with over 3400 cases and found that patients with co-deletion of chromosomal 1p/19q had a better PFS and OS than those with the entire chromosomal group (Zhao et al., 2014). MGMT promoter methylation is also a favorable molecular maker for a better clinical outcome in LGG (Mathur et al., 2020). In this paper, we found that patients with unmethylated MGMT promoter from the TCGA database tend to have a high-risk score, which suggests that MGMT promoter methylation may be related to the prognosis of LGG. However, the underlying mechanisms need to be explored in detail. In addition, since the number of cases in the TCGA database is still a bit small, the difference is not particularly obvious.

Several studies have explored the relationship between the expression changes of various genes and the development of glioma to identify appropriate prognostic markers for patients with glioma (Zhang et al., 2021b; Zhang et al., 2021c; Wang et al., 2021). Besides, with the rapid development of high-throughput sequencing technology, numerous gene families have been identified. Therefore, a series of reliable databases have been produced, such as the Gene Expression Omnibus (GEO), TCGA, and CGGA datasets. In the present study, we used two public databases (TCGA and CGGA) to investigate the correlation between the expression levels of the CMTM gene family and the prognosis of LGG.

The CMTM gene family was first described in 2001. The nine members in this family are located on different chromosomes, which play an essential role in various physiological and pathological processes (Hara et al., 1988). Delic et al. systematically analyzed CMTM family genes in GBM and found that CMTM2, 3, and 6 were significantly upregulated, and CMTM4 and 8 were significantly downregulated (Delic et al., 2015). They found that elevated CMTM1 and CMTM3 expressions were significantly associated with shorter OS. In addition, they provided first insights into CMTM1 and CMTM 3 signals through human phosphokinase protein expression profiling assay, which may be regulated by growth factor receptor, Src family kinase, and WNT activation. This perfect work inspired us to explore the role of CMTM family genes in LGG. CMTM1 and CMTM2, located on chromosome 16q21, mainly participate in chemotaxis and regulation of signaling receptor activity under physiological conditions. Previous studies showed that the expression of CMTM1 increased significantly in various cancer samples, suggesting that CMTM1 may play a vital role in tumorigenesis (Song et al., 2021). An *In vitro* study in a breast cancer cell line found that CMTM1 eliminated TNF-α-induced apoptosis and eventually promoted breast cancer cell proliferation (Wang et al., 2014). In this study, we found that the expression of CMTM1 was higher in grade III glioma in the TCGA datasets. Previous studies indicated that CMTM2 is downregulated in liver cancer tissues, which is related to the outcome of liver cancer patients (Li et al., 2020). However, no significant differences were found in the expression of various grades and genetic characteristics in LGG in our study.

CMTM3, CMTM4, and CMTM5 are thought to bring about favorable prognostic factors in several cancer types (Zhang et al., 2014; Hu et al., 2015; Li et al., 2015). Previous studies found that overexpressed CMTM3 significantly inhibited cancer cell proliferation and migration by reducing the activity of Erk1/2 (Su et al., 2014). However, in the present study, we found that upregulated CMTM3 may predict a poor outcome in LGG. In a recent paper on tumor cell proliferation and migration, the author found that over-expression of CMTM3 was associated with low pathological grade, high recurrence/metastasis rate, and worse survival in pancreatic cancer (Zhou et al., 2021). CMTM3 was found to closely relate to cell proliferation and differentiation, Hedgehog signaling pathway, Wnt signaling pathway, ECM-receptor interaction, and pathways in cancer. These results showed that the current role of CMTM3 in tumor formation is complex and ambiguous. CMTM3 may participate in tumor immunity through a variety of mechanisms, including signaling pathways that inhibit tumor growth and promote tumorigenesis.

CMTM4 can prevent cell proliferation and migration through the AKT/STAT3 pathway (Xue et al., 2019). *In vivo* and *In vitro* studies proved that CMTM5 could suppress tumor growth by regulating P13K-AKT signaling (Xiao et al., 2015; Xu and Dang, 2017). Our study confirmed that CMTM4 and CMTM5 are highly expressed in glioma with lower grade and better prognostic subtypes; however, the specific molecular mechanism warrants further investigation.

Intervention targeting immune checkpoints is an effective strategy in tumor immunotherapy. Recent reports have shown that CMTM6 regulates PD-L1, a key immune checkpoint in numerous cancers. CMTM6 effectively reduced the expression of PD-L1 through the IFN-γ signaling pathway (Burr et al., 2017; Mezzadra et al., 2017). Other CMTM members, including CMTM7 (Huang et al., 2019), CMTM8 (Gao et al., 2015), and CKLF (Dunne et al., 2016) were identified as tumor suppressors. Liu et al. found that CMTM7 plays a vista role in regulating EGFR signaling in human non-small cell lung cancer, and the knockdown of CMTM7 induces the progression of cancer cells (Liu et al., 2015). Further studies revealed that CMTM8 could induce cell apoptosis through a mitochondria-mediated pathway in caspase-independent and caspase-dependent manners (Li et al., 2014). CKLF, the first member to be discovered in the CMTM family, is also identified as a promising therapeutic target in human tumors (Cai et al., 2020). In this study, it was found that CMTM6, CMTM7, CMTM8, and CKLF were highly expressed in grade III and IDH-WT gliomas. These results indicated that these CMTM family members might have a different role in glioma than classical tumors.

Moreover, two clusters were established according to the expression of CMTM family genes, and a scoring model was built based on DEGs in the two clusters. It was found that the expression of CMTM3, CMTM6, CMTM7, CMTM8, and CKLF were significantly increased in cluster 2 and high-risk groups, and the expression of CMTM4 and CMTM5 was increased dramatically in cluster 1 and the low-risk group. The reliability of this model for predicting the prognosis of glioma was proved and then verified using the two validation datasets (CGGA-array and CGGA RNA-seq databases). The immune landscapes in the TME were investigated and found that different immune infiltrating cells were upregulated in cluster 2, such as DCs, B cells, CLPs, fibroblasts, macrophages, mast cells, Th2 cells, and monocytes, which play a vital role in tumor immunity. These results demonstrated that CMTM family genes are critical in the immune infiltration process of LGG TME.

Furthermore, GO and KEGG enrichment analyses were used to identify several enriched pathways, including cell cycle, NF-kappa B signaling pathway, p53 signaling pathway, PD-L1 expression, PD-1 checkpoint pathway, Th1 and Th2 cell differentiation, and TNF signaling pathway. These key pathways play a critical role in the process of tumor formation, development, and metastasis. Mounting evidence supports the role of the NF-kappa B pathway in the pathogenesis and resistance to treatment of glioma (Puliyappadamba et al., 2014). Especially, the common enriched pathways were p53 signaling pathway, PD-L1 expression, PD-1 checkpoint pathway, and Th17 cell differentiation based on the KEGG analysis. The p53 pathway is consisted of a network of genes and their products, which are designed to respond to various internal and external stress signals that affect the cell homeostasis mechanisms that monitor DNA replication, chromosome segregation, and cell division (Issaeva, 2019). Increasing studies have found that P53 signaling pathway can affect kinds of cellular processes, including maintenance of genome stability, metabolism, and longevity, and represents one of the most important and widely studied tumor suppressors (Stegh, 2012). Meanwhile, Th17 cells were identified as vital defenders against pathogens both in autoimmune disorders and cancer diseases (Knochelmann et al., 2018). In addition, PD-L1 and PD-1, which are negatively associated with immune checkpoint regulation, and have been proved to be predictive biomarkers of tumor immune therapy and are effective intervention targets in tumors (Chen et al., 2018). We found that PD-1, PD-L1, and PD-L2 expression levels were significantly upregulated in cluster 2 and high-risk groups in TCGA and CGGA databases. Importantly, the common enriched pathways were cell-cell adhesion mediated by integrin and regulation of antigen processing and presentation based on the GO analysis, which was found to take part in intercellular and cell-extracellular matrix interactions progress in multiple cancer types.

Generally, this study systematically described different expression patterns of CMTM family genes and their predictive value for patients with LGG. Furthermore, we explored the relationship between the expression of CMTM family genes and immune infiltrates in the TME. Overexpression of CMTM members is associated with crucial pathways implicated in tumor progression. The CMTM family can modulate LGG prognosis and tumor immunocytes infiltration even if the cluster model shows no statistical significance in multivariate Cox regression. But the scoring model is generated and constructed from the cluster model and shows better prognostic ability and more precise biofunction precision (Zhang et al., 2020). In sum, these findings underscore the importance of CMTM family genes as promising immunotherapeutic targets and could contribute to the discovery of novel immune checkpoints in LGG.

## Data Availability

The datasets presented in this study can be found in online repositories. The names of the repository/repositories and accession number(s) can be found in the article/Supplementary Material.
