# Supplementary material for: CMTM Family Genes Affect Prognosis and Modulate Immunocytes Infiltration in Grade II/III Glioma Patients by Influencing the Tumor Immune Landscape and Activating Associated Immunosuppressing Pathways
Source: Front Cell Dev Biol. 2022 Feb 17;10:740822. doi: 10.3389/fcell.2022.740822 (PMC8891612; doi:10.3389/fcell.2022.740822)
Supplement: Supplementary file 1 [file Presentation1.pdf]

Supplement Figure 1. Real-time quantitative PCR of CMTM family.

Real-time quantitative PCR of CMTM3, CMTM6, CMTM7, CMTM8 and CKLF. N=3. NS: no significance *p < 0.05. by the one way ANOVA test.

Supplement Figure 2. Different risk signatures of immune micro-environment in consensus clustering subgroups from training and validation cohorts

(A) Tumor purity in cluster 1 and cluster 2 from training and validation cohorts. (B) ESTIMATE score in cluster 1 and cluster 2 from training and validation cohorts. (C) Stromal score in cluster 1 and cluster 2 from training and validation cohorts. (D) Immune score in cluster 1 and cluster 2 from training and validation cohorts. ***p < 0.001; by the Wilcox test.

Supplement Figure 3. Immune cells infiltration in cluster 1 and cluster 2 from CGGA325 database

(A-C) CIBERSORT algorithm shows infiltrated immune cells in cluster 1 and cluster 2 from the TCGA (A), CGGA301 (B) and CGGA325 (C) database. *p< 0.05, **p < 0.01, ***p < 0.001, ns:no significant differences; by the Wilcox test.

Supplement Figure 4. Enriched pathways between cluster 1 and cluster 2 in CGGA325 database

(A) Enriched pathways between cluster 1 and cluster 2 based on KEGG-based GSEA analysis in the CGGA325 database. (B) Enriched pathways between cluster 1 and cluster 2 based on GO-based GSEA analysis in the CGGA325 database. (C) Enriched pathways between cluster 1 and cluster 2 based on GO-based GSVA analysis in the CGGA325 database.

Supplement Figure 5. Validation cohorts of GSE108474 databases.

(A) The CMTM expression in samples from GSE108474 and their corresponding clinical characteristic. Overall survival analysis based on the cluster model (B) and the scoring model (C).

Supplement Figure 6. The relationship between risk score and clinicopathologic features of LGG from training and validation cohorts

(A) Risk score among different IDH subtypes of LGG from the training and validation cohorts. (B) Risk score in LGG patients with different status of 1p/19q from the training and validation cohorts. (C) Risk score in LGG patients with different grades from the training and validation cohorts. (D) Risk score in LGG patients with different status of IDH from the training and validation cohorts. (E) Risk score in LGG patients with different status of MGMT from the training and validation cohorts. ***p < 0.001, ns:no significant differences; by the Wilcox test.

Supplement Figure 7. Enriched pathways between low and high risk groups in CGGA325 database

(A) Enriched pathways between low-risk and high-risk groups based on KEGG-based GSEA analysis in the CGGA325 database. (B) Enriched pathways between low-risk and high-risk groups based on GO-based GSEA analysis in the CGGA325 database. (C) Enriched pathways between low-risk and high-risk groups based on KEGG-based GSVA analysis in the CGGA325 database. (D) Enriched pathways between low-risk and high-risk groups based on GO-based GSVA analysis in the CGGA325 database.

	TCGA	CGGA-seq	CGGA-array	GSE108474
The number of samples	508	142	155	168
Grade<br>(Grade II, Grade III)	247, 260	93,49	111, 44	86, 82
Gender<br>(Female, Male)	225, 283	55, 87	63, 92	/
IDH<br>(Mutational, wildtype)	411, 94	104, 38	99, 54	/
1p19q<br>(codel. noncodel)	168. 340	54, 88	14, 23	/
MGMT<br>(Methylated, Unmethylated)	419.89	65, 65	37, 105	/
Gliovis subtype<br>(IDHmut-codel, IDHmut-noncodel, IDHwt)	190, 240, 78	63, 67, 12	50, 96, 9	37, 131, 0

Supplementary Table 1 | Description of samples from TCGA, CGGA-seq, CGGA-array database.

	TCGA				CGGA-seq				CGGA-array				GSE108474			
	Univariate Cox		Multivariate Cox		Univariate Cox		Multivariate Cox		Univariate Cox		Multivariate Cox		Univariate Cox		Multivariate Cox	
	P value	HR	P value	HR	P value	HR	P value	HR	P value	HR	P value	HR	P value	HR	P value	HR
Cluster	<0.001	3.375	0.097	0.623	<0.001	2.978	0.642	0.849	0.332	1.883	0.187	0.285	<0.001	1.879	0.362	1.209
Grade	<0.001	3.368	0.3	1.286	<0.001	3.018	<0.001	2.724	0.14	2.183	0.218	3.072	0.13	1.291	0.535	0.889
IDH	<0.001	6.641	0.048	0.449	<0.001	3.512	0.219	0.559	0.044	2.937	0.231	0.359	/	/	/	/
1p19q	<0.001	2.654	0.646	1.139	<0.001	5.583	<0.001	4.767	0.049	3.589	0.019	5.913	/	/	/	/
MGMT	<0.001	2.654	0.964	1.011	0.128	1.484	0.238	1.394	0.182	2.082	0.677	1.403	/	/	/	/
Score	<0.001	1.007	<0.001	1.009	<0.001	1.004	0.016	1.003	0.001	1.006	0.007	1.007	<0.001	1.003	<0.001	1.003

Supplementary Table 2 | Univariate and multivariate Cox regression analysis in TCGA, CGGA-seq, CGGA-array database and GSE108474.

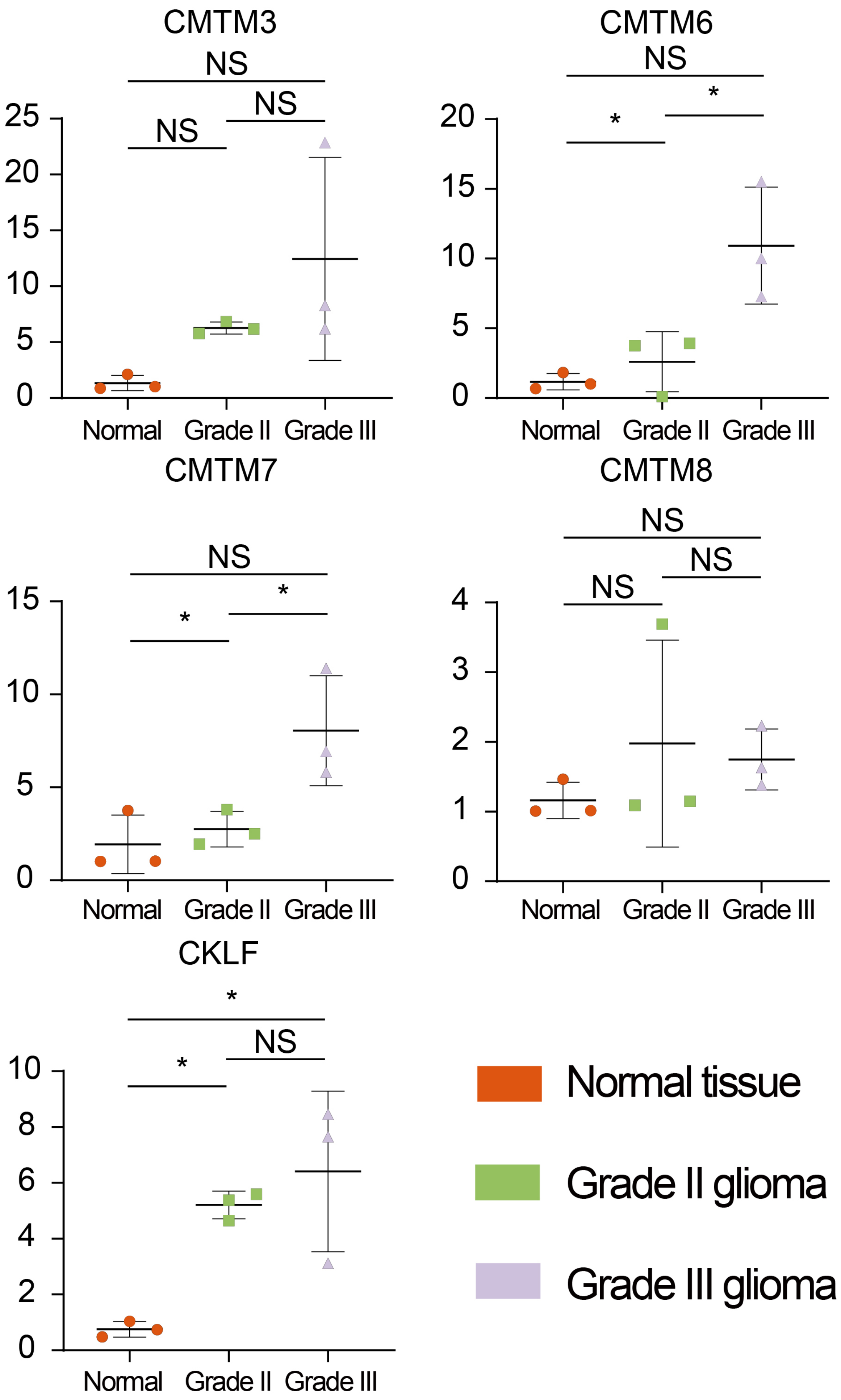

CMTM3
NS
NS
NS
25
20
15
10
5
0
Normal
Grade II
Grade III
CMTM6
NS
*
*
20
15
10
5
0
Normal
Grade II
Grade III
CMTM7
NS
*
*
15
10
5
0
Normal
Grade II
Grade III
CMTM8
NS
NS
NS
4
3
2
1
0
Normal
Grade II
Grade III
CKLF
*
NS
*
10
8
6
4
2
0
Normal
Grade II
Grade III
Normal tissue
Grade II glioma
Grade III glioma

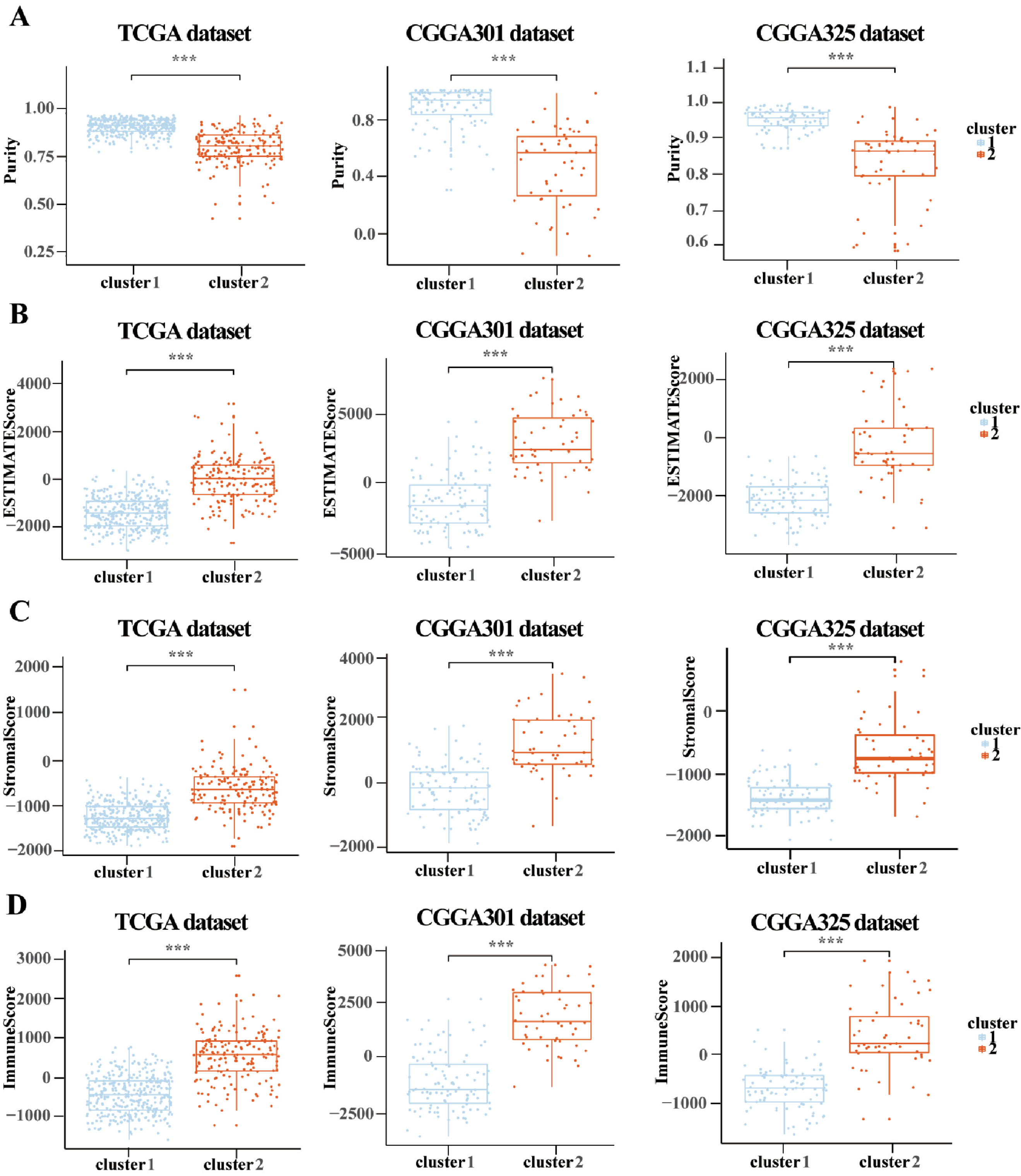

A
TCGA dataset
CGGA301 dataset
CGGA325 dataset
Purity
cluster 1
cluster 2
***
B
ESTIMATEScore
C
StromalScore
D
ImmuneScore
cluster
1
2

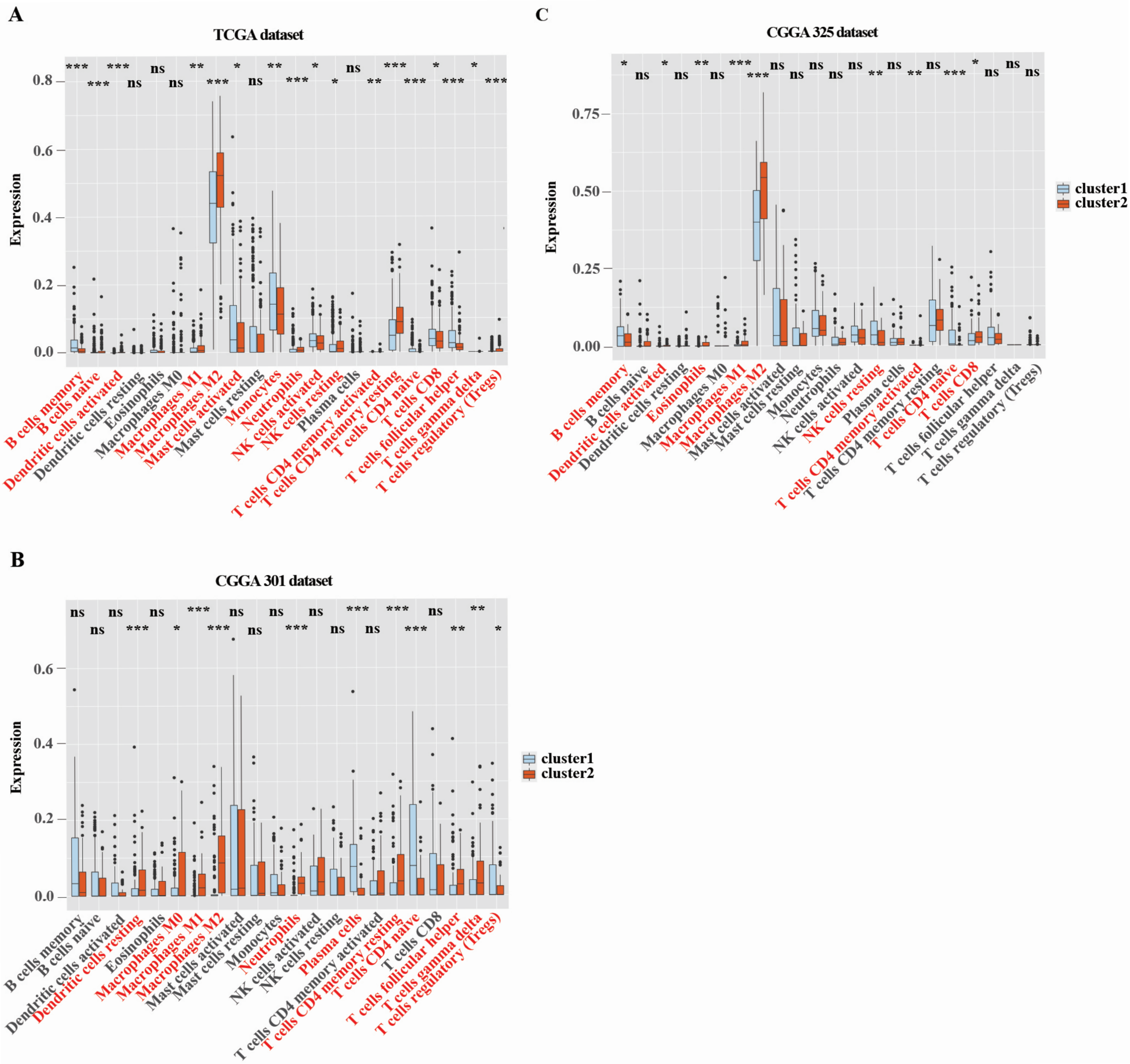
A
TCGA dataset
Expression
0.0
0.2
0.4
0.6
0.8
B cells memory
B cells naive
Dendritic cells activated
Dendritic cells resting
Eosinophils
Macrophages M0
Macrophages M1
Macrophages M2
Mast cells activated
Mast cells resting
Monocytes
Neutrophils
NK cells activated
NK cells resting
Plasma cells
T cells CD4 memory activated
T cells CD4 memory resting
T cells CD4 naive
T cells CD8
T cells follicular helper
T cells gamma delta
T cells regulatory (Tregs)
C
CGGA 325 dataset
Expression
0.00
0.25
0.50
0.75
cluster1
cluster2
B
CGGA 301 dataset
Expression
0.0
0.2
0.4
0.6
cluster1
cluster2

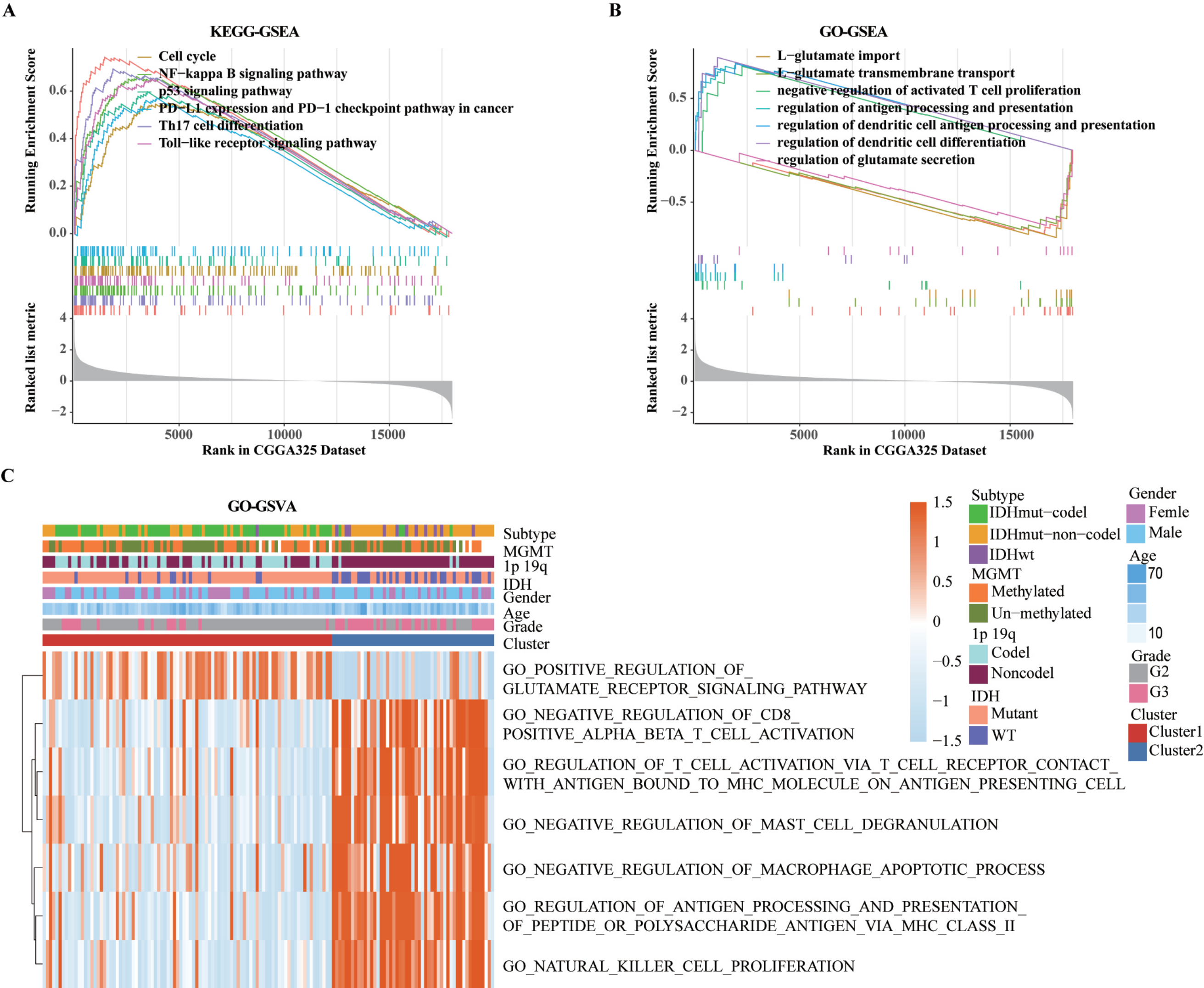
A
KEGG-GSEA
Cell cycle
NF−kappa B signaling pathway
p53 signaling pathway
PD−L1 expression and PD−1 checkpoint pathway in cancer
Th17 cell differentiation
Toll−like receptor signaling pathway
Running Enrichment Score
Ranked list metric
Rank in CGGA325 Dataset
B
GO-GSEA
L−glutamate import
L−glutamate transmembrane transport
negative regulation of activated T cell proliferation
regulation of antigen processing and presentation
regulation of dendritic cell antigen processing and presentation
regulation of dendritic cell differentiation
regulation of glutamate secretion
Running Enrichment Score
Ranked list metric
Rank in CGGA325 Dataset
C
GO-GSVA
Subtype
MGMT
1p 19q
IDH
Gender
Age
Grade
Cluster
GO_POSITIVE_REGULATION_OF_
GLUTAMATE_RECEPTOR_SIGNALING_PATHWAY
GO_NEGATIVE_REGULATION_OF_CD8_
POSITIVE_ALPHA_BETA_T_CELL_ACTIVATION
GO_REGULATION_OF_T_CELL_ACTIVATION_VIA_T_CELL_RECEPTOR_CONTACT_
WITH_ANTIGEN_BOUND_TO_MHC_MOLECULE_ON_ANTIGEN_PRESENTING_CELL
GO_NEGATIVE_REGULATION_OF_MAST_CELL_DEGRANULATION
GO_NEGATIVE_REGULATION_OF_MACROPHAGE_APOPTOTIC_PROCESS
GO_REGULATION_OF_ANTIGEN_PROCESSING_AND_PRESENTATION_
OF_PEPTIDE_OR_POLYSACCHARIDE_ANTIGEN_VIA_MHC_CLASS_II
GO_NATURAL_KILLER_CELL_PROLIFERATION
Subtype
IDHmut−codel
IDHmut−non−codel
IDHwt
MGMT
Methylated
Un−methylated
1p 19q
Codel
Noncodel
IDH
Mutant
WT
Gender
Femle
Male
Age
70
10
Grade
G2
G3
Cluster
Cluster1
Cluster2

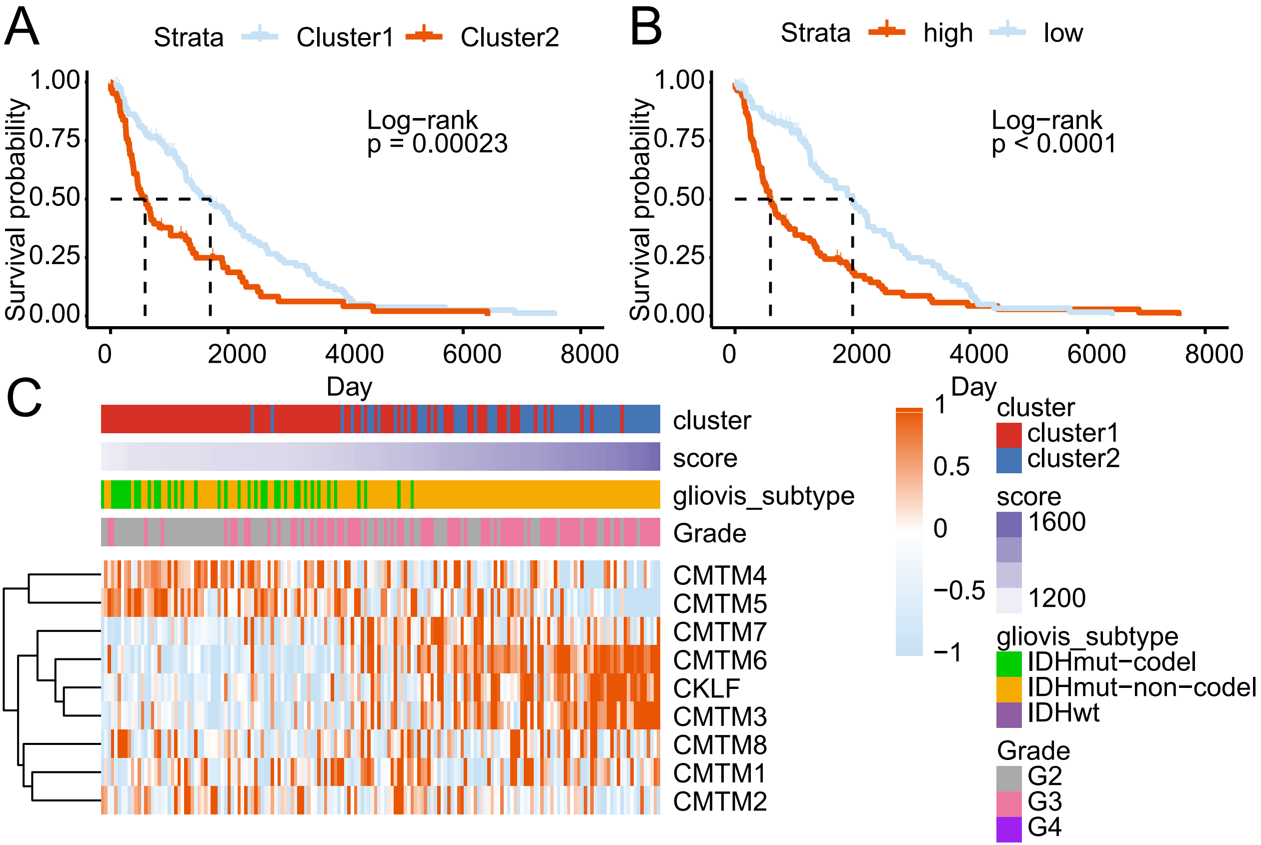
A
Strata
Cluster1
Cluster2
Survival probability
1.00
0.75
0.50
0.25
0.00
Log−rank
p = 0.00023
0
2000
4000
6000
8000
Day
B
Strata
high
low
Survival probability
Log−rank
p < 0.0001
Day
C
cluster
score
gliovis_subtype
Grade
CMTM4
CMTM5
CMTM7
CMTM6
CKLF
CMTM3
CMTM8
CMTM1
CMTM2
1
0.5
0
−0.5
−1
cluster
cluster1
cluster2
score
1600
1200
gliovis_subtype
IDHmut−codel
IDHmut−non−codel
IDHwt
Grade
G2
G3
G4

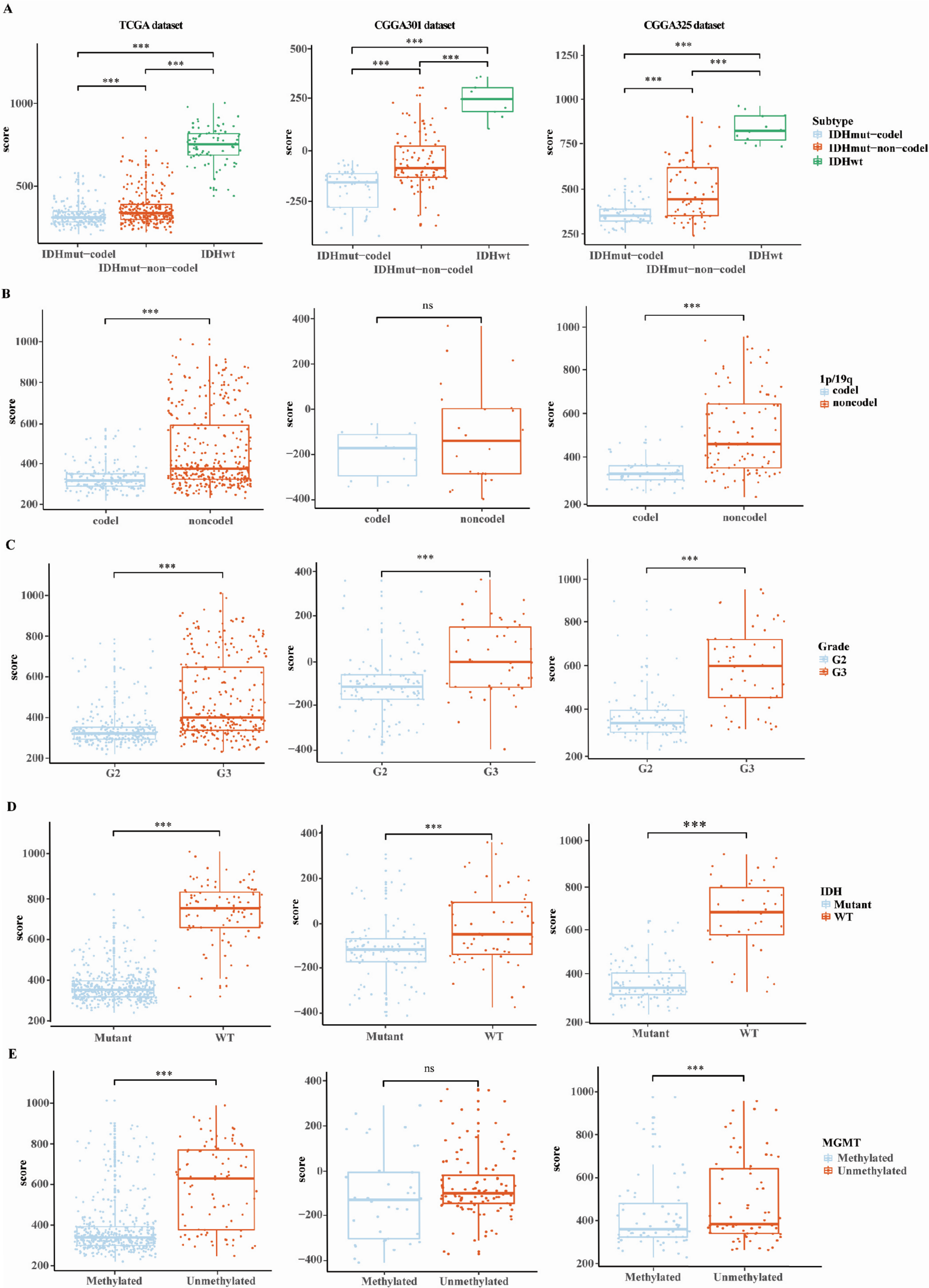

A
TCGA dataset
CGGA301 dataset
CGGA325 dataset
***
ns
score
IDHmut−codel
IDHmut−non−codel
IDHwt
Subtype
B
1p/19q
codel
noncodel
C
Grade
G2
G3
D
IDH
Mutant
WT
E
MGMT
Methylated
Unmethylated

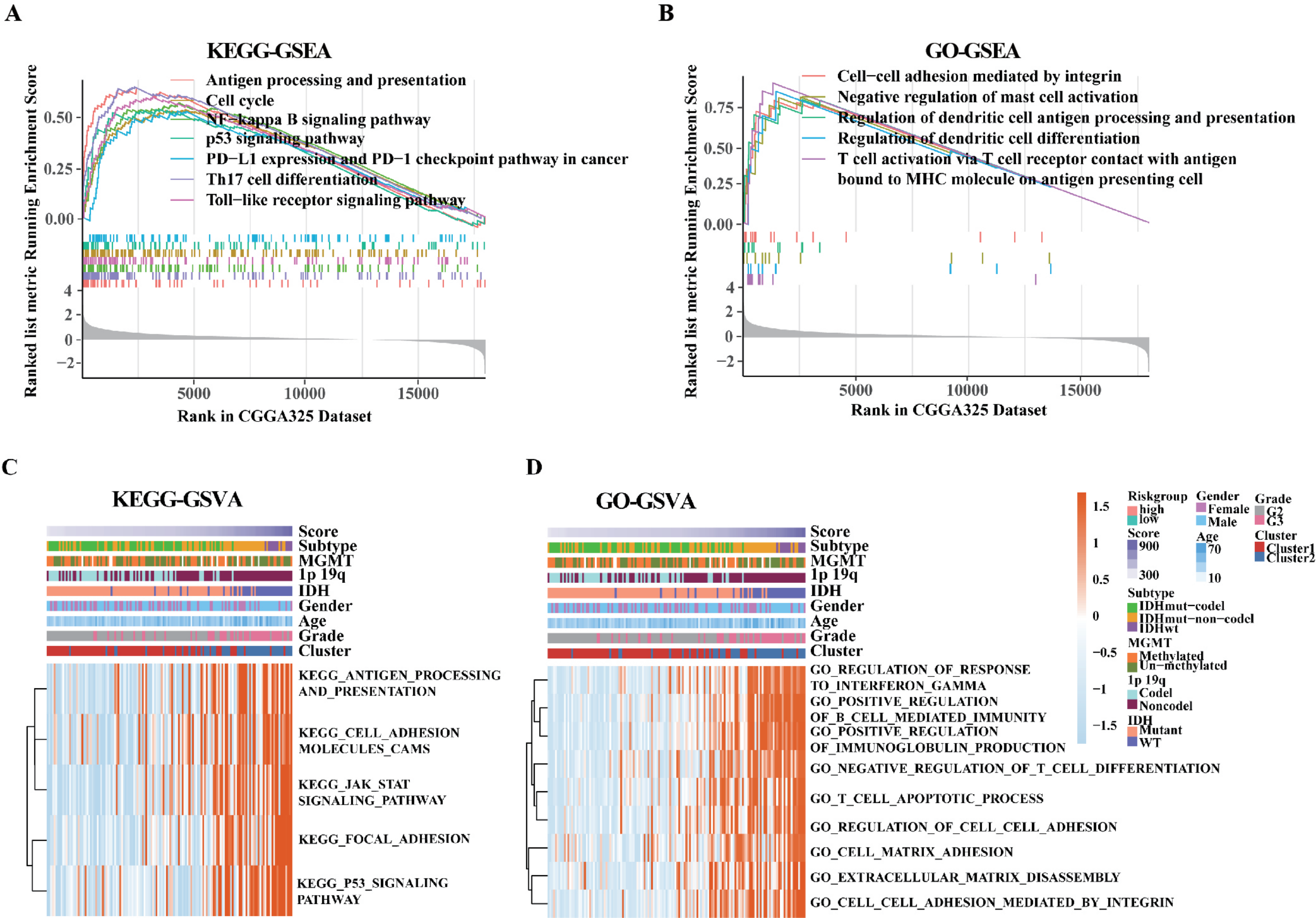
A
KEGG-GSEA
Antigen processing and presentation
Cell cycle
NF−kappa B signaling pathway
p53 signaling pathway
PD−L1 expression and PD−1 checkpoint pathway in cancer
Th17 cell differentiation
Toll−like receptor signaling pathway
Ranked list metric Running Enrichment Score
Rank in CGGA325 Dataset
B
GO-GSEA
Cell−cell adhesion mediated by integrin
Negative regulation of mast cell activation
Regulation of dendritic cell antigen processing and presentation
Regulation of dendritic cell differentiation
T cell activation via T cell receptor contact with antigen bound to MHC molecule on antigen presenting cell
Ranked list metric Running Enrichment Score
Rank in CGGA325 Dataset
C
KEGG-GSVA
Score
Subtype
MGMT
1p 19q
IDH
Gender
Age
Grade
Cluster
KEGG_ANTIGEN_PROCESSING AND_PRESENTATION
KEGG_CELL_ADHESION MOLECULES_CAMS
KEGG_JAK_STAT SIGNALING_PATHWAY
KEGG_FOCAL_ADHESION
KEGG_P53_SIGNALING PATHWAY
D
GO-GSVA
GO_REGULATION_OF_RESPONSE TO_INTERFERON_GAMMA
GO_POSITIVE_REGULATION OF_B_CELL_MEDIATED_IMMUNITY
GO_POSITIVE_REGULATION OF_IMMUNOGLOBULIN_PRODUCTION
GO_NEGATIVE_REGULATION_OF_T_CELL_DIFFERENTIATION
GO_T_CELL_APOPTOTIC_PROCESS
GO_REGULATION_OF_CELL_CELL_ADHESION
GO_CELL_MATRIX_ADHESION
GO_EXTRACELLULAR_MATRIX_DISASSEMBLY
GO_CELL_CELL_ADHESION_MEDIATED_BY_INTEGRIN
Riskgroup
high
low
Gender
Female
Male
Grade
G2
G3
Score
900
300
Age
70
10
Cluster
Cluster1
Cluster2
Subtype
IDHmut−codel
IDHmut−non−codel
IDHwt
MGMT
Methylated
Un−methylated
1p 19q
Codel
Noncodel
IDH
Mutant
WT
